# Supplementary material for: The interactome of intact mitochondria by cross-linking mass spectrometry provides evidence for coexisting respiratory supercomplexes
Source: Mol Cell Proteomics. 2017 Dec 8;17(2):216–32. doi: 10.1074/mcp.RA117.000470 (PMC5795388; doi:10.1074/mcp.RA117.000470)
Supplement: Supplemental Data [file supp_RA117.000470_133922_0_supp_23978_fzffwf.zip › spectra_annotation/mito_DR_spectra_annotation/95-1-3-1-7-1.pdf]

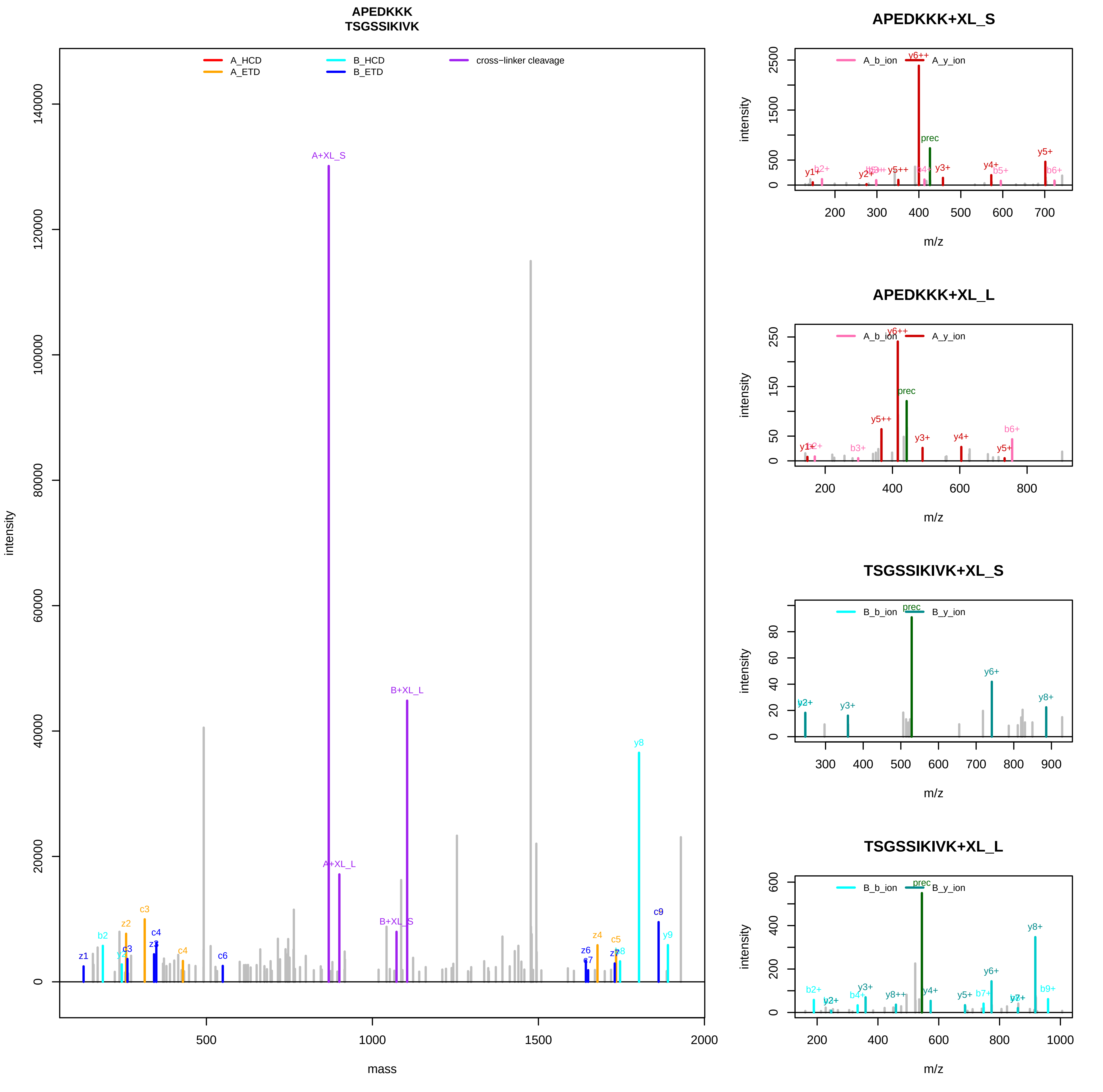
APEDKKK
TSGSSIKIVK
A_HCD
A_ETD
B_HCD
B_ETD
cross−linker cleavage
intensity
mass
A+XL_S
B+XL_L
A+XL_L
B+XL_S
z1
b2
z2
c3
c4
c6
z6
c7
z4
c5
z7
c9
y8
y9
APEDKKK+XL_S
APEDKKK+XL_L
TSGSSIKIVK+XL_S
TSGSSIKIVK+XL_L
A_b_ion
A_y_ion
B_b_ion
B_y_ion
prec
y6++
y5++
y1+
b2+
y2+
y3+
y4+
y5+
b3+
b4+
b5+
b6+
y6+
y8+
b9+
y8++
b7+
m/z
